# The Government mental hospital, Kilpauk, Madras: Memoirs of the fifties

**DOI:** 10.4103/0019-5545.43620

**Published:** 2008

**Authors:** P. Raghurami Reddy

**Affiliations:** Consultant Psychiatrist, Asha Hospital, House No. 3-5-783/13 A, Kingkoti, Hyderabad – 500029. E-mail: totalawareness@rediffmail.com

This is a commendable uncommon publication with lot of historical information, covering at depth various aspects of an Indian mental hospital from the perspective of its evolution, development, and administration. Choosing to write about this hospital was relevant for many reasons. Significantly, the hospital ‘is one of the largest in south Asia’, more than 200-years old. The author has rightly chosen and covered the activities of the hospital during 1950s, a turning point in the history of medical treatment of the mentally ill. It should interest intelligent lay persons and psychiatrists, particularly the younger generation, interested in historical aspects of development of a mental hospital services. The shrewdly selected 1950s was a crucial period of ‘chemical revolution’ in psychiatric management, when tranquilizers, antidepressants and anxiolytics were introduced. This had changed the face of mental hospitals, and ‘psychiatry became more humane’ and modern. As the author notes, “a recall of the history of this hospital reflects the evolution in the management of mental diseases in South India during the last two centuries.” – also true for India and many places in the world.

The first edition of 2004 had nearly 80 pages, dealing with the developments during 1950s. In the second edition of 2008, 50 more pages are added as part-II, dealing with various developments from 1957–2007. Thus, the book has become an updated publication of contemporary value. A publication on historical aspects is difficult to be flawless. The possible inaccuracies have been minimized by certain approaches.

The author worked in the hospital, for two decades, with three superintendents, from 1955–1978 with two gaps of total four years. First, for two years between 1956 and 1958, for postgraduate (PG) training in AIIMH, Bangalore; and the second one, for two years between 1960 and 1962, to UK under the Colombo plan. On his return, he started the speciality clinics, especially child guidance clinic. He is considered as ‘Father of child psychiatry’ in India. As a unique coincidence, the author's illustrious father was the President, Corporation of Madras, 1925 and was official visitor to this hospital.

Authenticity is added to the book with original articles of two persons who were associated with the development of the hospital in part-1; and articles of three superintendents in part-2. First article of part-1 is of Dr. N. Subramaniam, first RMO of the hospital, while the second article is of Dr. A. S. Johnson, superintendent from 1949–1957.

Part-2 has articles from three ex-superintendents: Dr. Sarada Menon,1961-1978; Dr. Ponnudurai, who has given a resume of the years from 1983–2007; and Dr. S. Nambi has covered comprehensively the role of the hospital in the activities of ‘The Indian Psychiatric Society’, appendix-III. Appendix-I has a list of illustrative deputy superintendents and medical officers of significance, with a note about them. Appendix-II has a useful write-up on Ancips-1957 conducted by the hospital.

The part-1 of the book has eleven chapters. Chapter-1, ‘The Beginnings of the Hospital’ is an extract from the book ‘Old Madras’ of Justice W. S. Krishnaswami Naidu, president of the hospital advisory committee, 1977–1981; has a short note on how the institution began. Chapters 2 and 3 have been taken from the souvenir of the hospital centenary celebrated in 1971. Chapter-2 is the article of Dr. N. Subramaniam, first RMO of the hospital, from 1954 for 16 long years. He had contributed a lot to the hospital in the areas of patient care and streamlining the administration. He has depicted a coherent history of the institution from its inception. The article has a detailed in-depth thorough description of the contributions of authorities, including the four superintendents he worked with. Chapter-3, the article of Dr. A. S. Johnson, superintendent 1949–1957, surveys the developments of that period. Chapters 4–11 are contributions of the author himself. Chapter-4 covers admission procedures, services organized, and varieties of treatments offered in 1950s. Chapters 5–10 gives an insight of infrastructural facilities and services provided in various wards and blocks, for different categories of patients like the ‘Back’ wards for chronically ill patients, women, criminal patients, mentally challenged, and mentally ill leprosy and tuberculosis (TB) patients. Chapter-11 describes the roles of the hospital staff – doctors, nurses, warders, etc., and quarters provided for staff.

Part-2 relates to 1957–2007, the next 50 years. The book has a thoughtful substantial contribution of 82 photos of the hospital blocks/wards, a ground plan of the hospital, and 29 photos of authorities up to 2007. The photographs are illustrative and educative, graphically reflecting the infrastructures and the activities of the hospital. Laborious effort must have gone into collecting them. Relevant photos of the blocks are suitably published at the end of each chapter. The hospital began functioning during the time of East India company, in 1794, as a modest private unit on a leased 45-acres land for 20 inmates and run by Dr. V. Connolly. Later Dr. Dalton, superintendent, rebuilt it, and had 54 inmates; it was popular as ‘Dalton's Mad Hospital’. In 1867, Government sanctioned a lunatic asylum on 66.5 acres of land. It functioned from 1871, having 145 inmates. In the year 1922, it was designated as ‘Government Mental Hospital’ in tune with the developments in psychiatric speciality; many developments followed thereafter. Dr. H. S. Hens man, superintendent 1924–1936, and Dr. G. R. Parasuram, deputy superintendent, improved the institution. They worked as a ‘dual team’ though had different temperaments. Dr. Hens man was a strict disciplinarian remembered for his morning hospital rounds as a ‘religious ritual’; and Dr. Parasuram builtup excellent tradition of doctor–patient relationship. Dr. Dhairyam, superintendent 1939–49, took a lead role in facilitating ‘voluntary board admissions’ that modified the application of the Indian lunacy act, 1912, encouraging many other psychiatrists and institutions in India to follow it. He introduced convulsive therapy, Insulin Coma therapy, and trials of indigenous medicines. Dr. A. S. Johnson, 1949–57, improved standards of patient care, planned numerous expansions and improvements, and rearranged wards. ‘Open hospital’ system was introduced, removing physical restraints. He introduced decentralization – with 13 medical officers, each one independently in charge of a ward. Psychiatric outpatient clinics were introduced in the two General hospitals of Madras, to promote referrals and reduce overcrowding in the mental hospital. Indian Red Cross Society started a 12-months course in social work at the hospital to improve patient care. Undergraduate (UG) teaching of psychiatry, for two weeks, was also started. Dr. T. George, superintendent 1957–61, provided new drugs, active treatment for all patients, a psychologist, and a separate new TB ward. With starting of AIIMH, Bangalore, Dr. Bushanam and Dr. O. Somasundaram were deputed for PG studies, who then returned in 1958.

Part-1: The author was himself a witness and contributor to many developments and could objectively describe the contents of the book in the chapters 4–11. Initially involuntary admissions later became more voluntary. Aggressive patients were isolated in single rooms. There were regularly 20–25 admissions. Treatment methods included pre-phenothiazine days, oral and parenteral barbiturates, paraldehyde, and chloral hydrate; Insulin coma therapy, Insulin histamine therapy for refractory schizophrenics, straight ECT's, injection Largactil;and serological test and LP for CSF examination in case of syphilitic patients. The ‘Back’ wards were for chronic cases. Care was custodial but humane.

There was often a need for physical restraints and isolation in single rooms. There were separate blocks for patients with common problems in long-term care. Blocks were named, the ‘Itch’ for scabies, and ‘Diarrhoea’, ‘Vigilance’ for suicidal and homicidal cases. Separate wards were organized for women, mentally abnormal offenders, and psychiatric patients with mental retardation, leprosy, and TB.

In the ward for criminal lunatics, impulsive and aggressive patients and cases of psychopathic temperament were also kept. On advice, the author injected two minims of turpentine to produce sterile abscess for the aggressive. The ward had a few cases of GPI, confirmed by serology and CSF with Lange's colloidal curve in the paretic, leutic forms. Penicillin replaced the arsenicals. For the mentally challenged cases, a ‘school’ was established. For Leprosy patients, expert's view was taken to separate the noninfective from infective cases. The author took the initiative and introduced antileprosy treatment – using chaulmoogra and hydnocarpus oils. For hypopigmented patches, intradermal ECCO injections were given, and antimony for lepromatous reactions. For TB cases, ‘sanatoria’ measures and treatment with Streptomycin, PAS, and Isoniacid were effective. Regarding the staff, the ‘superintendent’ had the key role, and was assisted by a deputy superintendent and medical officers. Initially, the attendants were designated as ‘warders’ – disturbed patients often manhandled. Things improved later, nurses were sanctioned and classes in psychiatric nursing introduced. After 1955, doctors and nurses were deputed for training in AIMH, Bangalore. Staff had quarters.

Part-2, 1957–2007: Dr. Sarada Menon, superintendent 1961–78. A three-unit system, in the outpatient and inpatient, was a dynamic useful change from the vertical system to horizontal system of shared work, ensuring continuity and better standards of patient care. New introductions were diagnostic procedures, clinical societies, refresher courses for general practitioners, medical library, departments of clinical psychology, EEG, and radiology. More medical and regular staff was sanctioned. Various administrative buildings were added including the outpatient block, and ‘day’ and ‘night’ hospital services started. Temporary discharge system was promoted to send many more inpatients home. By effectively screening admissions and improving care and discharge procedures, she could bring down the number of occupants from 2800 to 1800, the sanctioned strength. Teaching psychiatry to UG medical students was enhanced from two to four weeks, and teaching was offered to social workers from various universities. Teaching was assigned to medical officers and regular staff meetings ensured shared responsibility and interaction.

Also, part-2 of the book has an article on contribution of the author to the hospital development, as superintendent 1978–1983, besides his many-sided contributions from 1955–1978. The important events were: the beginnings of NMHP, increase in admission units from three to seven, MCI approval for Ph.D. training and for examinations in DPM and MD in psychiatry, hostel for PG students, two important ‘the Madras Longitudinal Study’ and ‘ICMR sponsored mutlicentric study’, course and outcome of schizophrenia, IPS conference 1982 in Madras, and release of the first Tamil book on psychiatry award winner “Psychiatry and its modern treatment” authored by Prof. O. Somasundaram and Dr. Jeyaramakrishnan.

In the chapter on ‘Introduction’, the author takes a stand against Western trend of downsizing mental hospitals and the fashion of ‘no more new mental hospitals’. Rightly, he advocates ‘well managed’ small institutions. He is in favor of Italian model with good backup of community mental health services; and the approach of Japan, which continues to keep ‘the so-called socially hospitalized patients’ for social reasons, and not for clinical reasons. The useful role of general hospital psychiatry units and need for mental hospitals in India for rehabilitation services for the severely mentally ill, unlike in the West, has been well noted. An insightful objection is taken about ‘unprecedented deinstitutionalization without community services’ in West, resulting in patients turned homeless; a case of human right violation. The author hopes that the mental hospitals in India will receive better funding and modernization. Truly, this hospital has a place of distinction in South Asia for high standard of PG training in psychiatry. This commendable and resource book deserves to be should be kept, as a ready reckoner, in all the psychiatric centers in India. Keeping its long-term use in mind, I would like it to be a bound edition.

